# The Application of Micro/Nanorobots in Cancer Therapy

**DOI:** 10.3390/mi17050612

**Published:** 2026-05-15

**Authors:** Yinglei Zhang, Bo Yang, Xiang Zou

**Affiliations:** 1 Postdoctoral Programme of Meteria Medica Institute of Harbin University of Commerce, Harbin 150076, China; susanp171221@163.com; 2 School of Pharmacy, Harbin University of Commerce, Harbin 150076, China; 3 Postdoctoral Station on Chinese Materia Medica, Harbin University of Commerce, Harbin 150076, China; 4 Engineering Research Center on Natural Antineoplastic Drugs, Ministry of Education, Harbin University of Commerce, Harbin 150076, China

**Keywords:** micro/nanorobots, cancer therapy, drug delivery system, tumor-targeting

## Abstract

Cancer continues to present a profound challenge due to high mortality and the inherent limitations of conventional treatments, including suboptimal targeting, systemic toxicity, and difficulty in overcoming physiological barriers. Micro/nanorobots (MNRs) offer a promising enhanced precision and efficacy in cancer therapy. This review systematically analyzes recent advancements in MNR applications, establishing a consistent framework that interlinks their diverse material compositions, propulsion strategies, and therapeutic functions. We critically compare various materials (inorganic, organic/polymeric, and biological/hybrid materials), elucidating their respective trade-offs in biocompatibility, biodegradability, and stimulus responsiveness. This paper further examines both internal (chemical and biological) and external (magnetic, light, and ultrasound) propulsion mechanisms, highlighting their strengths in overcoming biological barriers and enabling complex in vivo navigation, while also discussing their inherent limitations in control, fuel dependency, and tissue penetration. We then synthesize the therapeutic capabilities of MNRs across targeted drug delivery, phototherapy, radiotherapy, and immunotherapy, emphasizing common advantages like enhanced tumor specificity and reduced systemic side effects. A forward-looking perspective was also provided on the remaining challenges, particularly focusing on in vivo controllability, long-term biosafety, manufacturing scalability, and the significant hurdles in clinical translation. By offering a more critical and integrated analysis, this review underscores the immense potential of MNRs to revolutionize personalized precision cancer treatment, while candidly addressing the complex obstacles that must be surmounted for their successful clinical adoption.

## 1. Introduction

Despite significant advancements in traditional cancer therapies (e.g., surgery, radiotherapy, chemotherapy) that have enhanced patient survival and quality of life, these modalities often grapple with inherent limitations, such as insufficient precision, variable efficacy, severe systemic side effects, and collateral damage to healthy tissues. Furthermore, complex physiological barriers (e.g., the blood–brain barrier, the tumor microenvironment, and mucus layers) pose substantial obstacles to effective therapeutic delivery [1]. Consequently, recent research has increasingly focused on micro/nanobot technology as a novel therapeutic strategy to surmount these challenges. In order to overcome these issues, recent years have seen growing attention and research on micro/nanobot technology as a novel cancer treatment approach.

Micro/nanobots (MNRs) are miniature robotic systems designed for operation at the micro- or nanoscale. These entities enable precise navigation within the human body, facilitating sophisticated interventions such as targeted drug delivery, localized cancer cell destruction, and tumor tissue sampling and monitoring. Their advent offers a paradigm shift in cancer treatment, promising more precise and personalized therapies [2], mitigated side effects compared to conventional approaches, and enhanced therapeutic outcomes.

This review examines the applications of micro/nanobots in cancer treatment, delineating their mechanisms, design challenges, and preclinical studies. It further assesses their clinical advantages and limitations, culminating in a discussion of future prospects for this technology in oncology.

## 2. Integrated Design Strategies Based on Driving Mode

Bio-MNRs, despite their inherent biomedical advantages in targeted therapy and diagnostics, face considerable challenges within complex in vivo microenvironments. Dynamic physiological barriers, significant hydrodynamic resistance, and fluctuating local pH and oxygen levels critically impede their efficient locomotion and functionality [3,4]. To address these complexities and guide future design, this section establishes a comprehensive framework. It elucidates how diverse material properties are translated into active propulsion mechanisms, allowing for a systematic comparison of strategies for precise navigation and targeted intervention within complex biological milieus. This integrated approach not only describes various driving modes but also critically analyzes their interplay with material selection and their collective efficacy in overcoming physiological hurdles.

### 2.1. Internal Field Propulsion

**Figure 1:**
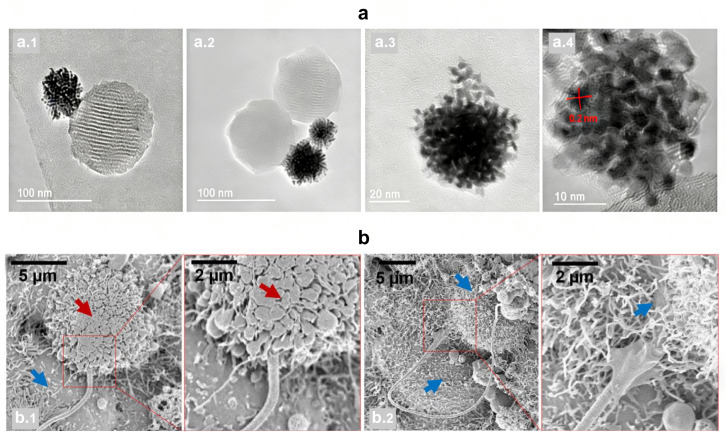
Examples of internal field propulsion mode micro/nanorobots. (**a**) High-resolution transmission electron microscopy images (HR-TEM) of Janus Pt-mesoporous silica nanoparticles (S_0_) (**a.1**,**a.2**) and PtNds (**a.3**,**a.4**), showing the interplanar spacing of 2.3 Å in the (111) plane of PtNd crystals [5], Copyright 2021, American Chemical Society; (**b**) SEM images showing the sperm-HeLa cell fusion. (**b.1**) Cell fusion with the doxorubicin hydrochloride-loaded sperm; (**b.2**) cell fusion with an unloaded sperm. Red arrows point at a cell in apoptosis and the blue arrows point at live cells [6], Copyright 2017, American Chemical Society.

As shown in Figure 1, MNRs propelled internally by chemical or biological mechanisms, hold immense potential in cancer therapy for targeted drug delivery, precise diagnosis, and minimally invasive interventions. Chemical propulsion offers high thrust and autonomy but is limited by fuel availability and potential toxicity, while biological propulsion provides biocompatibility and inherent targeting capabilities, though it faces challenges with immune response, safety, and control complexity. Despite these hurdles, MNRs offer unique advantages in overcoming physiological barriers, enhancing drug concentration at tumor sites, and reducing systemic toxicity.

#### 2.1.1. Chemically Propelled MNRs (C-MNRs): Mechanisms, Applications, and Comparative Analysis

Chemically propelled MNRs (C-MNRs) leverage ambient chemical energy to achieve autonomous mobility and functional versatility, finding applications in sensing, environmental remediation, and targeted therapeutics [7,8]. A fundamental engineering principle for C-MNRs is asymmetric design, which localizes chemical reactions to specific sites on the robot surface rather than uniform activity [5,9]. This spatial segregation, often manifested in Janus structures with distinct active and inert hemispheres [10,11], is crucial for generating localized product release or ion gradients that drive propulsion. C-MNR propulsion mechanisms can be broadly categorized into bubble-driven, concentration gradient-driven (self-diffusiophoresis), and self-generated electric field-driven (bipolar electrochemistry). Each mechanism offers distinct advantages and faces unique limitations, dictating their suitability for different biological applications.

##### Bubble-Driven Propulsion

Bubble-driven propulsion relies on the rapid generation and expulsion of gas bubbles (e.g., O_2_, H_2_, and NO) through the catalytic decomposition of fuels like H_2_O_2_ or stomach acid. The resulting swift, non-reciprocal impulse provides substantial thrust, making these systems highly effective in overcoming high-viscosity barriers, such as mucus, and establishing them as some of the fastest chemical robots with strong thrust, and effective penetration of viscous environments. Meanwhile, the jerky, discontinuous motion can hinder precise navigation. Furthermore, the generation of gas bubbles can be problematic in certain in vivo contexts due to potential embolism or localized tissue damage, and the fuel availability (e.g., high H_2_O_2_ concentrations) might be limited in healthy tissues. For instance, zinc-based robots utilize gastric acid to generate H_2_ bubbles for oral drug delivery and acid neutralization. Mg-based Janus micromotors have delivered Curcumin through mucus layers in ulcerative colitis [12]. Importantly, many C-MNRs exploit the tumor microenvironment (TME), which is often rich in H_2_O_2_ due to abnormal metabolism and acidic pH. By loading catalysts like platinum (Pt), manganese dioxide (MnO_2_), or catalase, these nanobots efficiently decompose H_2_O_2_ into water and oxygen, generating powerful oxygen bubble recoil forces that enable efficient navigation through viscous tumor tissue [13]. Wan et al. engineered doxorubicin-loaded nanomotors (HFLA-DOX construct) that exhibit intrinsic motion and sustained nitric oxide (NO) release, aiming to enhance drug penetration and reverse multidrug resistance in cancer chemotherapy [14]. This approach highlights the dual functionality of propulsion and therapeutic agent release.

##### Concentration Gradient-Driven Propulsion

In environments with dissolved reaction products, C-MNRs can propel themselves via self-diffusiophoresis. This mechanism establishes a neutral solute gradient around the C-MNR’s active site, where the interaction between these solutes and the C-MNR surface generates a pressure imbalance, propelling the robot away from higher concentrations. This mechanism typically results in smoother, more continuous motion compared to bubble-driven systems, making it potentially more suitable for delicate navigation. Their high sensitivity to local molecular environments renders them particularly suitable for biosensing applications. It has slower propulsion speeds compared to bubble-driven systems, and its efficiency is highly dependent on the stability and magnitude of local concentration gradients. For example, Ji et al. demonstrated a glucose-powered Janus nanomotor functionalized with glucose oxidase and polydopamine-Fe^3+^ chelates. When immersed in a glucose solution, the glucose oxidase catalyzes glucose decomposition, creating a localized glucose concentration gradient that propels the motors at a notable speed (2.67 µm/s). This active diffusion enhances cellular uptake, facilitates Fe^3+^ ion delivery, and H_2_O_2_ generation, thereby boosting ferroptosis and inducing potent cancer cell death [15].

##### Self-Generated Electric Field-Driven Propulsion

Chemical triggers instigate electron–hole separation, with subsequent redox reactions generating localized ion concentrations (e.g., H^+^ or Cl^−^). The differential diffusion of these ions establishes a self-generated electric field, which acts upon the robot’s zeta potential. Specifically, C-MNRs operating in the Charge Separation on Both Sides mode function as bipolar electrochemical cells. This is achieved by integrating source and sink electrodes on opposite ends of a single nanostructure, thereby creating a continuous internal flow of electrons. This electron flow drives oxidation at one end and reduction at the other, resulting in a net ion flow from head to tail. This mechanism offers sophisticated directional control, primarily by manipulating Fermi levels or applying weak external directional control fields to bias the internal chemical potential. It allows for more precise manipulation in complex microfluidic environments. Therefore, it requires specific material properties for efficient electron–hole separation and may be sensitive to changes in ionic strength or conductivity of the biological medium. Liang et al. demonstrated the hierarchical self-organization and collective behaviors of dissimilar microparticles driven by local electrohydrodynamic and diffusiophoretic interactions. The application of an AC electric field induced dissimilar dielectric microparticles (varying in size or dielectric properties) to hierarchically self-organize into leader–follower microswarms through electrohydrodynamic interactions [16]. This indicates potential for collective, coordinated movement for enhanced therapeutic efficacy.

Beyond material composition, robot shape significantly dictates motility. Tubular designs, for instance, facilitate gas bubble accumulation and jet-like ejection, achieving greater speeds than spherical counterparts. Recent advancements in 3D nano-printing and template-assisted electrodeposition enable complex geometries such as spirals, bowls, and matchstick shapes, which optimize fluidic drag and rotational stability, thereby enhancing overall propulsion efficiency and stability. While C-MNRs offer autonomous operation, challenges remain in controlling their precise trajectories and speeds in heterogeneous in vivo environments. Fuel depletion, potential cytotoxicity from reaction byproducts, and the difficulty in real-time external manipulation limit their broader clinical applicability. Future research needs to focus on biodegradable materials, biocompatible fuels, and integrating sensing capabilities for adaptive navigation, mimicking the memory behavior of chemotactic microorganisms as demonstrated by Mou et al. [17].

##### Nanozymes: Versatile Components for Advanced C-MNR Design

Nanozymes present a platform for nanorobot development, attributed to their distinct attributes such as adaptable designs, tunable enzyme-like functions, and nanoscale physicochemical characteristics. Their utility extends to participation within single systems or integration across multiple nanorobot frameworks [18]. Illustratively, Wang et al. engineered a biomimetic nanoflower via nanozyme self-assembly. This innovation catalyzes a cascade of intracellular biochemical reactions, generating reactive oxygen species (ROS) under both normoxic and hypoxic conditions, critically, without requiring external stimuli. Beyond its capacity to mitigate hypoxic states, this system robustly induces cell apoptosis through a ROS-mediated mechanism, thereby achieving significant and specific tumor growth inhibition [19].

In recent years, design strategies for functional nanomotors have exhibited a trend toward diversification, with core research centering on energy conversion mechanisms, bio-interface interactions, and potential clinical applications. In the study of fundamental kinetics, Arque et al. [20] investigated the intrinsic relationship between enzyme kinetics and self-propulsion using a combined approach of experimental analysis and molecular dynamics simulations. By immobilizing urease, acetylcholinesterase, glucose oxidase, and aldolase onto silica microcapsules, they systematically analyzed how enzyme turnover numbers and conformational dynamics precisely drive the movement of these micromotors. In the realm of biomedical applications, researchers have focused on enhancing the biological effects of nanomotors through multi-enzyme integration. One research group integrated multiple enzymes onto the surface of Janus nanoparticles; the resulting synergistic motion not only enhanced receptor-mediated endocytosis but also promoted exocytosis via the endoplasmic reticulum/Golgi apparatus pathway, significantly increasing the internalization efficiency of the nanoparticles into tumor cells [21,22]. Furthermore, to address the size constraints hindering clinical translation, the team developed a universal and scalable colloidal chemistry synthesis method. They successfully fabricated ultrasmall urease-powered Janus nanomotors with diameters ranging from 30 to 100 nm, specifically designed to effectively overcome various biological barriers within the bloodstream [23].

#### 2.1.2. Bio-Hybrid Driving

Bio-hybrid drives represent a biomimetic strategy that integrates inherently motile microorganisms or living cells as engines for nanorobots. These systems typically involve attaching drug-loaded nanoparticles to the surface of these biological entities, which maintain their natural flagella/cilia propulsion and sophisticated sensing systems. This approach fundamentally leverages the evolved capabilities of biological systems to achieve active propulsion, targeting, and biocompatibility, offering a distinct advantage over purely synthetic methods in navigating complex biological environments.

##### Bacteria-Based MNRs

Bacteria, such as magnetotactic bacteria and Salmonella, are natural microbots endowed with efficient flagellar propulsion, sensitive chemosensing systems, and surface receptors for nutrient acquisition and environmental interaction via chemical cues [24,25,26]. Anaerobic bacteria exhibit natural chemotaxis towards and cluster within the hypoxic core of tumors, making them ideal vehicles for anti-cancer drug delivery. Researchers engineer bacterial robots by affixing drug-loaded nanoparticles to attenuated bacterial surfaces. These robots leverage their flagella for rapid motility and employ chemotaxis to precisely accumulate in hypoxic tumor regions [27,28]. Bacteria-based cancer vaccines can acquire attributes like prolonged efficacy, enhanced targeting, self-adjuvancy, and on-demand cargo release by emulating inherent biological properties such as stealth coatings, pathogen recognition patterns, and tissue tropism [29]. While highly effective in navigating to hypoxic tumor regions, potential issues include immune response to the bacterial component, ensuring precise attenuation to prevent pathogenicity, and controlled drug release kinetics. The complex in vivo environment can still pose challenges for their long-term stability and precise control.

##### Virus-like Particles (VLPs)

VLPs, formed by the self-assembly of viral capsid proteins, offer an efficient strategy for drug and gene carriage [30]. They retain the inherent cellular invasion and genetic material delivery efficiency of viruses while lacking infectious genetic material, significantly enhancing their safety profile [31,32]. This makes them attractive for targeted delivery without the pathogenicity of live viruses. But VLPs typically lack intrinsic propulsion and rely on passive diffusion or external forces for navigation. Their primary advantage lies in targeted delivery and cellular internalization, rather than active locomotion. The specificity of targeting can also be influenced by the presence of host antibodies.

##### Cell-Based MNRs

Cellular-driven MNRs (e.g., sperm-hybrid micromotors [6], neutrophil-integrated microcrafts [33,34]) leverage the inherent motility and targeting abilities of cells to carry therapeutic payloads. Sperm offers rapid propulsion in fluidic environments (e.g., female reproductive tract), while neutrophils provide immune evasion and active targeting of inflammatory sites [33]. The primary advantage is high biocompatibility and reduced immune response, often accompanied by complex biological functions. Yet, complex challenges involve achieving efficient and stable cell-nanoparticle conjugation, maintaining cell viability and function over prolonged periods, and developing precise remote control mechanisms that do not interfere with natural cellular processes [6,33].

##### DNA Origami-Based MNRs

DNA origami MNRs represent a distinct bio-hybrid approach, employing programmable self-assembly of DNA strands to create complex, responsive nanostructures [35]. They offer highly precise conformational changes triggered by specific biological signals such as miRNA, pH, enabling smart drug release or activation. Their modular design allows for versatile therapeutic functions and excellent biocompatibility [36,37]. The main limitations include potential degradation by nucleases in vivo, complex large-scale fabrication, and relatively slower propulsion mechanisms compared to other active systems, often requiring integration with other propulsive elements [38,39,40]. The precision of their programming is a key strength, but their kinematic limitations are a notable trade-off.

### 2.2. External Field Propulsion

**Figure 2:**
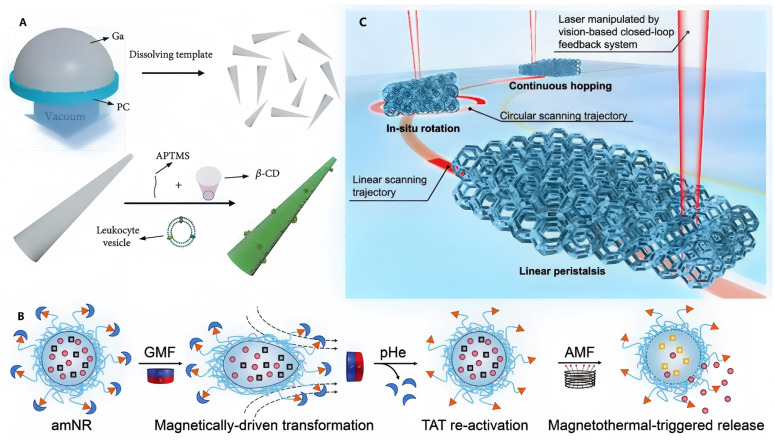
Examples of external field propulsion mode micro/nanorobots. (**A**) Schematic illustration of the fabrication process of leukocyte membrane-coated gallium nanoswimmers (LMGNSs) [41], Copyright 2020, American Association for the Advancement of Science. (**B**) The schematic diagram illustrates the light-driven lattice soft microrobots (LSMR) in the application scenario of multimodal locomotion [42], Copyright 2025, Springer Nature. (**C**) Schematic of the structural changes in amoeba-like nanorobot (amNR) under the application of a magnetic field [43], Copyright 2023, Wiley-VCH.

External field propulsion, as shown in Figure 2, offers a powerful paradigm for remotely controlling MNRs, providing precise manipulation and fuel-free operation without the need for internal power sources. This approach overcomes key limitations of internal propulsion by enabling real-time, on-demand control over robot movement within complex biological environments.

#### 2.2.1. Magnetic Field Propulsion: Precision Navigation and Theranostic Integration

Magnetic field driving is a cornerstone of external field propulsion, employing magnetic nanoparticles embedded within nanorobots. Their inherent superparamagnetism confers two critical advantages for MNRs: (1) Precise Remote Magnetic Navigation: Under an applied magnetic field gradient, Fe_3_O_4_ nanorobots can be accurately directed to any physiological depth [8,44]. This capability is crucial for surmounting challenges posed by blood flow and ensuring efficient active targeting in deep tissues [45,46]. Rotating magnetic fields enable spiral nanorobots to drill into viscous tissues, while gradient magnetic fields guide nanorobot swarms to collectively overcome physiological obstacles. This is particularly promising for difficult-to-access areas like the blood–brain barrier penetration [47]. (2) MRI Contrast Enhancement and Theranostic Integration: Fe_3_O_4_ serves as an effective T2-weighted MRI contrast agent, enabling real-time in vivo tracking of MNRs position, distribution, and enrichment during drug delivery. This dual diagnostic–therapeutic (theranostic) capability provides a visual foundation for personalized precision treatment [48]. For dense tissues or fluids, the clustering strategy generates greater output force, enabling smoother navigation on uneven surfaces and overcoming high-resistance environments [49]. The extremely small planar geometric structures achieved through nanolithography technology are crucial for minimizing immune responses and enabling efficient clearance [50].

Soft MNRs, capable of intricate deformations including elongation, contraction, and bending, are fabricated by embedding magnetic micro/nanoparticle fillers such as neodymium iron boron and ferroferric oxide within compliant polymer matrices like Ecoflex, polydimethylsiloxane, or hydrogel. These composites respond dynamically to external magnetic fields [51,52]. A key advancement in their design involves encoding specific internal magnetization profiles, often achieved through unidirectional magnetization technology applied to pre-folded origami structures. This process, which entails magnetizing the actuators to saturation within a strong unidirectional magnetic field, results in magnetization profiles precisely aligned with the applied field’s direction [53].

Despite the immense potential, fabricating magnetically controllable nanorobots of suitable size for complex in vivo drug delivery remains a significant challenge. Issues include off-target accumulation, potential heating effects from strong magnetic fields [54], and the complexity of external magnetic actuation systems required for precise 3D control. While remote control is a major advantage, ensuring the nanorobots remain localized at the target site without undesired dispersion after the magnetic field is removed, is crucial for safety and efficacy.

#### 2.2.2. Light Propulsion

Photo-drive utilizes photosensitive materials to convert light energy into kinetic energy, offering spatial and temporal resolution, enabling manipulation at the cellular level. This mechanism is particularly valuable for applications where high precision is paramount. Light propulsion mechanisms are primarily categorized into two types: The photothermal effect involves materials such as gold nanorods generating localized temperature gradients under near-infrared (NIR) light irradiation, thereby driving their motion through the thermophoreic effect. The photochemical effect relies on semiconductor materials, which can catalyze the decomposition of surrounding liquids (such as hydrogen peroxide solutions) under ultraviolet or visible light, generating localized chemical gradients or bubbles, thereby exerting its effects through chemical propulsion [6]. Such optically controlled motion offers excellent spatiotemporal controllability, enabling precise manipulation. However, the main bottleneck of light driving is its significantly limited tissue penetration depth [55]. Ultraviolet light, in particular, has very shallow penetration, restricting its widespread application in deep tissues. Consequently, light-driven MNRs are primarily suitable for superficial diseases like skin cancer or luminal lesions accessible by endoscopes (e.g., colon cancer). Furthermore, some photochemical propulsion mechanisms still require the presence of specific chemical fuels (e.g., H_2_O_2_), which may not be uniformly available in all in vivo environments.

To overcome the penetration depth limitation, innovative approaches like negative phototaxis have emerged. That is, the MNRs exhibit light-avoiding motion, actively moving away from external light sources towards deep tumor regions. This mechanism critically breaks through the passive diffusion limitation of traditional nanoparticles, allowing for deeper engagement [56,57]. Moreover, light-driven nanorobots loaded with photosensitizers and anti-cancer medicines can actively gather around cancer cells and produce photothermal-induced hyperthermia and photosynthetic oxygen to modulate immunity [58], which could simultaneously achieve photothermal treatment in synergy with chemotherapy.

Photodrive is the use of photosensitive materials to convert light energy into kinetic energy, providing spatial and temporal resolution, enabling manipulation at the cell level. Mechanisms and applications are mainly divided into two categories: one is based on photothermal effects, such as gold nanorods generating local thermal gradients under near-infrared light irradiation, and driving their own motion through thermophoresis; the other is based on photochemical effects with semiconductor materials. Such as titanium dioxide (TiO_2_), a prominent semiconductor photocatalyst, primarily serves as a light-driven engine in nanorobotics. When configured asymmetrically (e.g., in Janus structures with platinum), TiO_2_, under ultraviolet or visible light irradiation, catalyzes the decomposition of ambient liquids (e.g., hydrogen peroxide solution), generating local chemical gradients or bubbles. This subsequently induces active nanobot motion via self-diffusion or bubble recoil. Such optically controlled motion offers spatiotemporal controllability, enabling precise manipulation. However, its widespread application in deep tissue is significantly limited by the shallow penetration depth of driving light (especially ultraviolet light) and the requisite presence of specific chemical fuels (e.g., H_2_O_2_) [59]. Novel hydrogels, particularly those prepared from poly(N-isopropylacrylamide) (PNIPAM) monomers combined with photo-initiators and photo-cross-linkers, exhibit immense potential. These hydrogels leverage their temperature-sensitive properties and environmental responsiveness, achieving precise responses to light stimuli through the incorporation of photothermal conversion materials like gold nanoparticles [60]. Upon laser irradiation, these dopants efficiently absorb light energy and convert it into heat, triggering localized contraction and deformation of the hydrogel, thereby enabling microrobot actuation. In cancer therapy, these hydrogels are engineered as carriers for microspheres or nanoparticles. Temperature changes induced by the photothermal effect are utilized to precisely control the drug release rate, allowing for targeted and quantitative drug delivery [61].

#### 2.2.3. Ultrasonic Propulsion

Ultrasonic propulsion capitalizes on ultrasound’s inherent deep tissue penetration and excellent biocompatibility, establishing it as a compelling external power source for MNRs. Microrobots with specific asymmetric structures achieve stable directional motion by exploiting unbalanced acoustic radiation forces in an ultrasonic standing wave field. Ultrasound waves exert radiation forces that can propel micro/nanorobots, providing precise and controllable movement. Its cavitation effect instantaneously increases cell membrane permeability, facilitating simultaneous nanorobot guidance to target cells and synergistically promoting anticancer drug endocytosis. This is a significant advantage for enhancing therapeutic uptake at the cellular level. Ultrasonic-driven MNRs have been successfully applied in targeted delivery in viscous environments, such as the vitreous cavity and joint cavities [41]. By modulating the ultrasound frequency and voltage, active targeting, penetration, and internalization of cancer cells can be achieved [41].

Polymeric materials have emerged as highly versatile tools in ultrasound imaging and therapy, offering superior control over physicochemical properties compared to traditional lipid or protein-based agents. These materials serve as cavitation nuclei, enhancing ultrasound imaging resolution and facilitating therapeutic applications like localized drug delivery and transient biological barrier opening. Key polymer types, including PBCA, PMAA, PMMA, PVA, PVP, PLGA, and PVAX, are utilized to engineer diverse ultrasound-responsive platforms such as microbubbles, nanocups, microcapsules, and gas-releasing nanoparticles. The design principles leverage differences in acoustic impedance, either by stabilizing gas bubbles within shells or cavities, generating bubbles in situ, or creating rigid multilayered structures. Furthermore, the properties of polymeric microbubbles can be precisely tailored by manipulating their shape, shell thickness, stiffness, and bioconjugation profile, which in turn influences their circulation time, drug loading capacity, and acoustic response. These engineered polymeric microbubbles are finding advanced applications in ultrasound imaging, targeted molecular imaging and drug delivery, therapeutic gas release, blood–brain barrier (BBB) sonopermeation, and other ultrasound-mediated therapies like antibacterial activity and transdermal drug penetration. Beyond biomedical uses, they also show promise in non-medical fields such as optical displays and adhesive removal [62].

While ultrasonic propulsion offers excellent tissue penetration and biocompatibility, challenges remain in achieving highly precise, sub-cellular level control, especially in heterogeneous tissue environments. The energy deposition needs to be carefully controlled to avoid adverse effects from cavitation or heating on healthy tissues. Compared to magnetic fields, which offer robust force, ultrasound can be more diffuse in its energy delivery, making fine-grained control more complex. However, its non-invasiveness and ability to enhance membrane permeability provide a unique synergistic advantage, particularly for drug delivery applications. Future research will focus on developing smarter polymeric materials and advanced ultrasound control algorithms to achieve even finer spatial and temporal precision.

### 2.3. MNRs in Cancer Therapy: A Framework of Materials, Propulsion Strategies, and Therapeutic Functions

MNRs represent a transformative technology in cancer therapy, addressing critical limitations of conventional treatments through their ability to precisely navigate complex in vivo microenvironments, deliver therapeutic agents, and perform targeted interventions. This framework provides a comprehensive overview of MNRs, categorizing them by their constituent material types, including inorganic materials (shown in Table 1), organic/polymeric materials (shown in Table 2) and biological/hybrid materials (shown in Table 3). It highlights the intricate interplay between design choices and functional outcomes, supported by relevant research findings.

## 3. Cancer Treatment Strategies with MNRs

**Figure 3:**
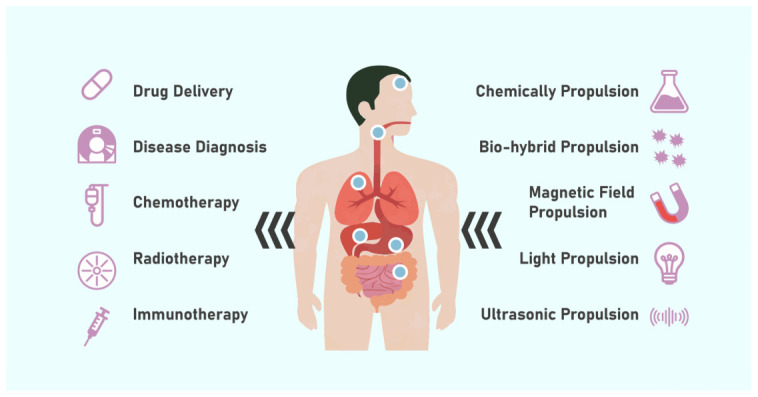
Schematic view of micro/nanorobots in cancer therapy.

As shown in Figure 3, MNRs offer substantial potential in cancer treatment, extending beyond precise drug delivery to encompass the optimization of existing therapeutic methods. Their indispensable role is increasingly acknowledged across diverse facets of cancer management, including tumor-targeted therapy, enhancing radiotherapy and chemotherapy, facilitating immunotherapy, and integrating with emerging treatment strategies.

### 3.1. Strategies for Enhanced Drug Delivery and Barrier Penetration Based on MNRs

Traditional cancer treatments are often limited by a lack of precision, hindering specific drug targeting to tumor sites [63] and causing collateral damage to healthy tissues. MNRs offer a transformative solution by enabling precise navigation within the body to directly target tumor tissues, thereby minimizing systemic side effects and enhancing therapeutic outcomes.

#### 3.1.1. Targeting and Multi-Modal Activation

Chen et al. [64] developed intelligent cell-derived nanorobots that not only improved tumor accumulation and cancer cell uptake via cell membrane camouflaging but also responded to ultrasound stimuli. This dual action enhanced intratumor blood flow and oxygen supply, effectively overcoming treatment barriers and activating sonodynamic proptosis. This system exemplifies the synergistic benefit of combining biological camouflage with external field activation (ultrasound for deep penetration and triggered release), a key strategy for overcoming complex in vivo barriers.

Kang et al. fabricated EA@BTO_microrobots, incorporating BaTiO_3_ and encased in enteric microcapsules, produced via photocurable 3D printing [65]. These microrobots provide protection during digestion, facilitate tumor targeting, enable mucus penetration, and mediate gas release, ensuring precise, responsive delivery within the intestinal tract’s anaerobic and acidic environments. Upon reaching the tumor, the integrated BaTiO_3_ nanoparticles catalyze reduction–oxidation reactions under ultrasound irradiation, inducing immunogenic tumor cell death. Moreover, both BaTiO_3_ and EA consume lactic acid, alleviating the tumor’s immunosuppressive microenvironment, promoting dendritic cell maturation and M1 macrophage polarization, and reducing regulatory T cell populations. This sophisticated design highlights the trend towards multi-functional MNRs that not only deliver drugs but also actively modulate the tumor microenvironment for enhanced therapeutic effect, a significant advancement over passive drug carriers.

#### 3.1.2. Overcoming the Blood–Brain Barrier (BBB)

**Figure 4:**
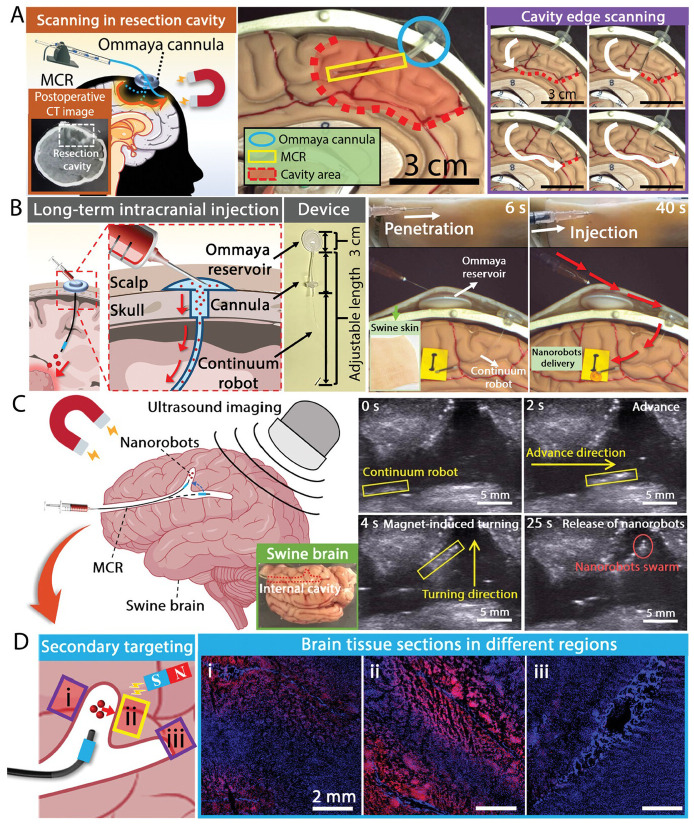
Intracranial cross-scale targeting drug delivery of marsupial robotic system. (**A**) Scanning the edge of the simulated resection cavity in a one-to-one scale model of the human brain using MCR. **Left**: schematic illustration, **inset**: CT image of resection cavity in clinical practice. **Right**: Experimental images. (**B**) Long-term administration of the marsupial robotic system through the Ommaya device. **Left**: schematic illustration. **Right**: Experimental images. (**C**) Ultrasound imaging-assisted targeting transport of nanorobots inside the swine brain using MCR. **Left**: Schematic illustration. **Right**: Experimental images. (**D**) Secondary targeting of nanorobots in the swine brain. **Left**: Schematic illustration. **Right**: Fluorescence images of brain tissue sections derived from different regions (marked i–iii). Red: Nanorobots labeled with Rhodamine B, blue: cell nuclei stained with DAPI [67], Copyright 2024, Wiley-VCH.

The BBB represents a formidable obstacle, blocking over 98% of therapeutic agents from accessing intracranial neoplastic lesions [66]. As shown in Figure 4, engineered nanorobots (<200 nm) leverage diverse transport mechanisms, including receptor- and carrier-mediated pathways, to bypass the BBB. This approach substantially enhances targeting and therapeutic efficacy while mitigating systemic toxicity. As shown in Figure 4, Wu et al. introduced a marsupial robotic system for hierarchical drug delivery [67]. This system comprises a magnetic continuum “mother robot” that navigates the BBB at the macroscale to reach pathological sites, and then deploys hybrid “child nanorobots” that achieve microscale precision targeting. This hierarchical strategy markedly enhances both targeting accuracy and therapeutic efficacy for localized intracranial therapeutics, representing a critical advancement for brain tumor treatment by combining macro-scale navigation with micro-scale precision. Liu et al. presented an allosteric DNA nanorobot for targeted glioma therapy, engineered via DNA origami for acid-triggered doxorubicin (Dox) delivery [68]. This nanorobot not only effectively crosses the BBB but also accumulates in glioma tissues, resulting in significant suppression of tumor growth. The programmability of DNA origami allows for highly specific, TME-responsive drug release, offering a precise and adaptable solution for challenging targets like brain tumors.

MNRs offer significant advantages over conventional systemic chemotherapy by delivering drugs locally, which not only reduces side effects but also increases drug concentration and bioavailability at the tumor site. The strategies we have discussed ranging from cell-derived camouflaging to hierarchical systems and responsive DNA origami demonstrate a clear trend towards increasingly intelligent and multi-functional designs. While preclinical models show promising therapeutic outcomes, challenges remain in ensuring long-term in vivo stability, avoiding off-target accumulation, and developing robust, real-time control systems for precise navigation in dynamic biological environments. The integration of intelligent feedback mechanisms with advanced navigation systems is crucial for realizing the full potential of MNRs in precision drug delivery.

### 3.2. MNRs in Cancer Diagnosis: Based Imaging, Towards Intelligent Biosensing

MNRs not only facilitate precise drug delivery but also enable complex intratumoral tasks such as cellular manipulation and real-time tumor progression monitoring. While conventional nanomaterial-based contrast agents have advanced diagnostic imaging, deep tissue access remains a significant challenge [69]. MNRs, as promising contrast agent carriers, demonstrate significant advancements in facilitating highly efficient bioimaging for disease diagnosis by overcoming these limitations.

#### 3.2.1. Multi-Functional Sensing and Visualization

Li et al. [69] designed swarming-responsive photonic nanorobots for dual functions: mapping local physicochemical conditions and facilitating the motile-targeting visualization of unknown targets (e.g., tumor lesions) to guide external NIR light for localized photothermal treatment. This system integrates sensing, active targeting, and therapeutic guidance, offering a comprehensive diagnostic and interventional platform.

**Figure 5:**
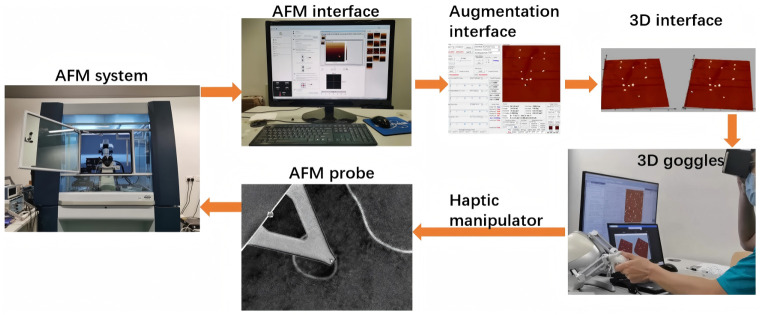
Schematic of the stereoscopic vision-based augmentation reality nanorobotic system [70], Copyright 2024, Springer Nature.

The advanced AFM-based nanorobotic platform shown in Figure 5 integrates an augmented reality framework that dynamically refreshes topographical imagery via real-time force feedback during active operations [70]. For extraordinarily delicate procedures, such as physical manipulation of viral particles, mechanical interactions at the probe tip remain within a narrow threshold of piconewtons to nanonewtons. A specialized haptic controller translates these infinitesimal stimuli into tangible tactile sensations for the human operator. This technology addresses the micro–macro gap, enabling human operators to feel and manipulate nanoscale entities, a critical advancement for high-precision diagnostic and interventional procedures.

Perfluorocarbon-encapsulated magnetic nanocarriers can be deployed under an external alternating magnetic field [71]. These carriers effectively transduce electromagnetic energy into heat, triggering a droplet vaporization process critical for targeted ultrasound imaging. Because the spatial reach of the applied magnetic field is extensive enough to envelop entire organs, this energy conversion mechanism ensures highly precise, macro-scale phase-change imaging. This approach provides deep-tissue diagnostic capabilities by leveraging a physical phase change triggered by an external field, offering a distinct advantage over optical methods.

#### 3.2.2. Enhanced Intracellular Biomarker Detection

Sensitive and rapid imaging of intracellular cancer-associated miRNA holds significant potential for early cancer diagnosis and treatment monitoring. Conventional nanoparticle-based imaging probes, however, often rely on passive diffusion, leading to prolonged response times and reduced target recognition due to solution viscous resistance. To address this, Wu et al. developed a DNA tetrahedral-modified magnetic nanorobot [72]. This nanorobot enabled surface-localized framework nucleic acid-located catalytic hairpin assembly. The tetrahedral structure imparted exceptional structural stability and efficient cell-uptake performance. Concurrently, its spatial confinement effect expedited and optimized hairpin cascade signal amplification. Importantly, the probe’s active movement enhanced fluidic micromixing and accelerated target capture, thereby significantly reducing reaction time and improving reaction kinetics. This strategy generated enhanced fluorescence signals and precisely discriminated between miR-21 and various other miRNA sequences, confirming its feasibility and versatility across normal and diverse cancer cell lines. This system critically demonstrates how active propulsion can overcome diffusion limitations, significantly enhancing the speed and sensitivity of intracellular biomarker detection, a crucial step for early diagnosis.

MNRs offer a significant leap forward in cancer diagnosis by enabling active targeting, real-time feedback, and enhanced reaction kinetics, which are often lacking in passive nanoparticle-based systems. While magnetic fields provide deep tissue penetration for imaging, optical methods offer higher resolution for superficial targets [73]. The integration of haptic feedback and active movement in biosensors allows for unprecedented precision in manipulation and detection. The trade-off often lies between penetration depth, spatial resolution, and the complexity of the control system. Future research will focus on developing multi-modal diagnostic MNRs that combine deep-tissue imaging with high-resolution sensing, as well as integrating more sophisticated on-board intelligence for autonomous decision-making and data analysis, ultimately leading to more precise and personalized cancer diagnosis and therapy.

### 3.3. MNRs in Cancer Phototherapy: Enhancing Targeted Light-Activated Therapies

Phototherapy, a noncontact diagnostic and therapeutic modality, has gained widespread medical adoption due to robust technological advancements. Its mechanism, which utilizes specific wavelengths of light to activate photosensitive materials, facilitates non- or minimally invasive treatments in vivo and in vitro. This approach offers exceptional selectivity and localization, significantly mitigating collateral damage often associated with conventional therapies. Phototherapy broadly encompasses two main modalities: photodynamic therapy (PDT) and photothermal therapy (PTT). The integration of MNRs with phototherapy has pioneered the field of light-driven or light-responsive MNRs, significantly augmenting targeting efficacy and therapeutic precision, thus minimizing harm to healthy tissues [74].

#### 3.3.1. Photodynamic Therapy (PDT) with MNRs

**Figure 6:**
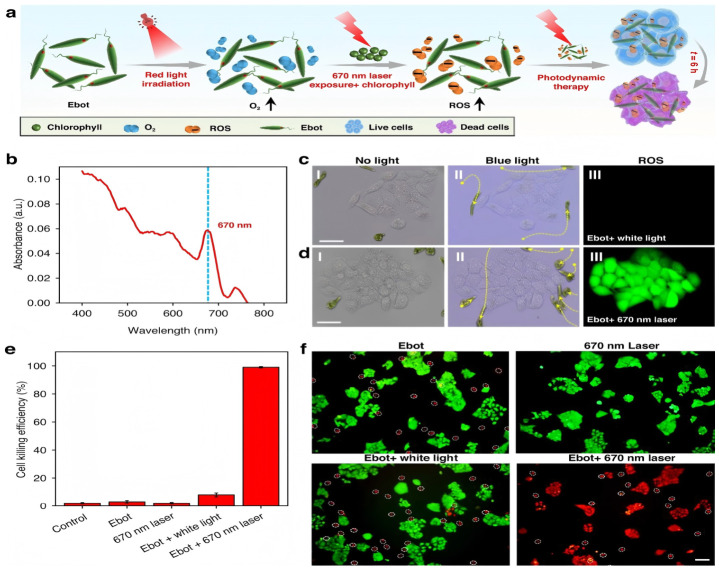
Ebot for photodynamic therapy (PDT). (**a**) Schematic illustration of PDT using Ebot. (**b**) Light absorption of EG. (**c**,**d**) Experimental results showing Ebot navigation and ROS generation. (**I**,**II**) Microscopic images showing navigation of Ebot to designated locations with blue light irradiation, (**III**) Fluorescent images showing the generation of ROS (green fluorescence) with different light irradiation. (**e**) Histogram showing the efficiency of PDT under different treatments of Ebot. (**f**) Fluorescent images showing the results of PDT with different treatments of Ebot. HeLa cells stained with Calcein-AM (green, live cells) and PI (red, dead cells), the white dashed circle represents Ebots. Scale bar: 50 μm. Data for (**e**) are presented as mean values ± s.d. (*n* = 16) [76], Copyright 2024, Springer Nature.

PDT involves the synergistic action of a photosensitizer, a specific wavelength of light, and oxygen. Upon light absorption in the tumor region, the photosensitizer is activated, generating highly reactive singlet oxygen (^1^O_2_) and other reactive oxygen species (ROS). These ROS are cytotoxic to cancer cells, leading to cellular damage and death. Biocatalytic Cascade-Driven PDT could be conducted by integrating an artificial enzyme with upconversion nanoparticles and zirconium/iron porphyrin metal–organic framework core–shell nanoparticles [75]. This system leverages the efficient ROS generation capabilities of enzymes, combined with upconversion properties for deeper light penetration, which is a significant improvement over traditional PDT by enhancing the localized generation of ROS and overcoming shallow light penetration. As shown in Figure 6, light-controlled soft biomicrorobots based on Euglena gracilis are capable of performing multiple tasks in narrow microenvironments, including intestinal mucosa, with high controllability, deformability, and adaptability [76]. These robots can pass through narrow and curved microchannels, execute tasks such as targeted drug delivery, selective removal of diseased cells in intestinal mucosa, and photodynamic therapy. This highlights the versatility of biologically derived MNRs to adapt to complex anatomical structures while simultaneously delivering PDT. PDT often requires a sufficient oxygen supply in the tumor microenvironment, which can be hypoxic. While some MNR designs aim to enhance oxygen, this remains a challenge. The penetration depth of activating light is also a limiting factor, especially for deep-seated tumors.

#### 3.3.2. Photothermal Therapy (PTT) with MNRs

PTT utilizes photothermal agents (e.g., gold nanorods, copper sulfide, graphene, and polyaniline) that efficiently absorb near-infrared (NIR) light and convert the light energy into heat, locally raising tumor temperature (typically >42 °C). This leads to irreversible damage or death of cancer cells. NIR light offers relatively good tissue penetration compared to visible or UV light [77].

**Figure 7:**
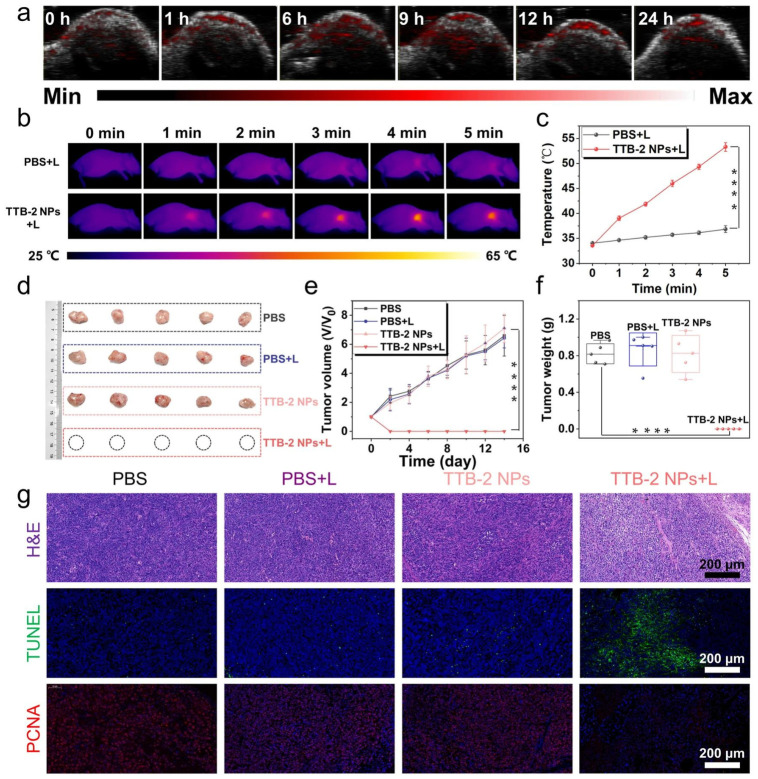
(**a**) In vivo PA imaging of the tumor at different times of TTB-2 NPs post-injection. (**b**) Representative thermal images and (**c**) corresponding temperature profile of the tumor in various treated mice. (**d**) Tumor isolated from the mice after various treatments. (**e**) Relative tumor growth curves of different treatments as a function of treated time. (**f**) Tumor weight changes in different treatments at 14 days. (**g**) H&E, TUNEL, and PCNA staining of tumors separated from the treated mice. (**** *p* < 0.0001, n = 5) [78], Copyright 2023, Springer Nature.

Swarming-Responsive Photonic MNRs for Localized PTT could dynamically map local physicochemical conditions and facilitate motile-targeting visualization of unknown targets (e.g., tumor lesions), subsequently guiding external NIR light for localized photothermal treatment [69]. This active targeting capability significantly enhances the precision of PTT, ensuring that heat is generated specifically at the tumor site, minimizing damage to healthy tissues. As shown in Figure 7, Gao et al. demonstrated that D-A conjugated diradical polymer TTB-2 nanoparticles (NPs) achieved effective tumor suppression and photoacoustic imaging in vitro and in vivo when illuminated with NIR-II light [78]. The use of advanced photothermal agents with NIR-II light offers deeper tissue penetration and more efficient heat conversion, leading to improved therapeutic outcomes for deep-seated tumors. While NIR light penetrates deeper, precise temperature control in heterogeneous biological tissues can be challenging to avoid off-target thermal damage. Continuous, real-time monitoring of local temperature is crucial but complex.

Both PDT and PTT benefit significantly from integration with MNRs by enabling active targeting, precise localization, and synergistic therapeutic effects. PDT is highly effective in generating cytotoxic ROS, but it is limited by oxygen availability and light penetration. PTT offers deep penetration with NIR light and direct cell killing via hyperthermia but requires precise temperature management. The choice between PDT and PTT, and their integration with specific MNR designs, often depends on the tumor type, its location, oxygenation status, and desired depth of penetration. Future research could focus on developing multi-modal MNRs that combine PDT and PTT, potentially with drug delivery, to overcome individual limitations and achieve superior therapeutic efficacy. Improving in vivo real-time monitoring of therapeutic effects and minimizing off-target damage remain critical challenges. Other research groups indirectly indicated that the inflammatory signal triggered by photothermal therapy can maintain a recruitment effect of neutrophils for up to further days. Similar ideas have also been applied to radiotherapy for gastric cancer, which increased the release of inflammatory factors while performing tumor destruction [79].

### 3.4. MNRs in Supporting Radiotherapy: Overcoming Resistance and Enhancing Precision

Radiotherapy (RT) is a primary treatment modality for many cancers, including early non-resectable lung cancer. While RT effectively inhibits tumor growth by inducing DNA damage, its efficacy is severely hampered by intratumoral hypoxia and the presence of radioresistant cancer stem cells [80]. Hypoxia-induced physiological shifts not only bolster cancer cell survival mechanisms but also facilitate neovascularization, creating a protective shield against radiation-induced oxidative stress. This hostile tumor microenvironment (TME) fosters a resilient niche that often evades conventional treatment. MNRs offer a promising nanotechnology-based solution to overcome these intrinsic limitations, primarily by neutralizing hypoxia, reversing radioresistance, and enhancing the radiotherapeutic impact on the tumor mass [81].

**Figure 8:**
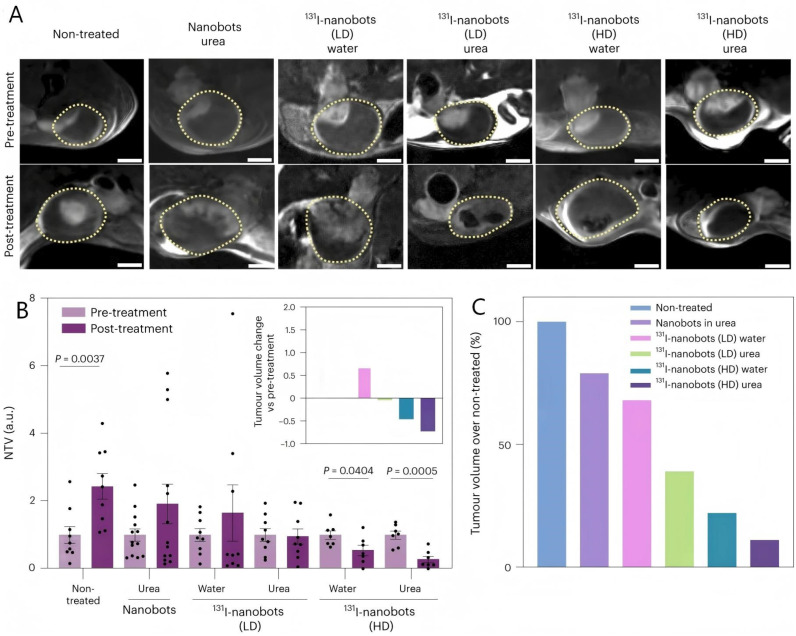
(**A**) DW-MRI 2D slices through the bladder of tumor-bearing mice before and after treatment with radionuclides; low-dose (LD) and high-dose (HD) denote 1.85 MBq and 18.5 MBq doses of ^131^I, respectively; yellow dotted lines show the bladders. Scale bars, 2 mm. (**B**) NTV obtained by MRI before and after treatment. Tumor volumes were normalized by the means of the pretreatment values of each group (n = 9 per group, except n = 13 for nanobots in urea and n = 7 for high-dose ^131^I-nanobots in water and urea, biological replicates). Data are presented as mean values and error bars represent the s.e.m. Statistical significances are based on a two-tailed unpaired *t*-test. Inset: Tumor volume changes with respect to pretreatment (see (**C**) for legend). (**C**) Post-treatment tumor volumes normalized to the control condition (non-treated group) [82], Copyright 2024, Springer Nature.

MNRs have certain advantages in radiotherapy, which can be designed to deliver oxygen-generating agents or to modulate the TME to alleviate hypoxia, thereby increasing the radiosensitivity of tumor cells. This is a critical advantage, as oxygen is essential for the formation of DNA-damaging free radicals during RT. They also could facilitate localized radiation delivery directly to tumor sites, thereby minimizing collateral exposure to healthy tissues and mitigating common radiotherapy adverse effects like skin damage and immune suppression. As shown in Figure 8, Sánchez et al. [82] developed an innovative urea enzyme-driven nanorobot for the treatment of bladder cancer. This system was loaded with radionuclides (^18^F and ^131^I) for both diagnostic imaging and radiotherapy. This approach demonstrates how MNRs can precisely deliver radionuclides to the tumor, leveraging enzymatic propulsion for controlled navigation, which is a significant improvement over systemic radionuclide administration. Although initial reservations existed regarding the precision and stability of MNRs, recent advancements in magnetic field control and automated navigation systems have markedly enhanced their therapeutic stability and targeting accuracy. This technological progress is crucial for realizing the full potential of personalized, precise cancer treatment with MNRs in conjunction with radiotherapy. Beyond direct radionuclide delivery, some MNRs can modulate the TME, for example, by reducing lactic acid or activating immune responses, which can further sensitize tumors to radiation or enhance the overall anti-tumor immune response.

MNRs provide a significant advantage over conventional radiotherapy by offering precise delivery of radiosensitizers or radionuclides, and by actively modulating the adverse TME conditions like hypoxia. The integration of MNRs allows for a multi-pronged approach that targets tumor cells, sensitizes them to radiation, and protects healthy tissues. However, challenges remain in ensuring the precise deposition of radiation dose, especially with actively moving robots, and in assessing the long-term biological effects of combining nanorobots with radiation. Future research needs to focus on real-time dosimetry, integrating advanced imaging for feedback-controlled navigation during RT, and developing biodegradable MNRs that can be safely cleared from the body after treatment.

### 3.5. MNRs in Immunotherapy

By mimicking cell membrane structures, MNRs can effectively prolong the in vivo half-life of drugs, specifically accumulate at tumor sites, and simultaneously regulate local immune responses [83]. Within this framework, several innovative approaches have emerged: leveraging the natural tumor-homing and thrombus-forming properties of platelets, researchers have developed MNRs to deliver immune checkpoint inhibitors (e.g., aPD-1). These MNRs either induce local thrombosis at the tumor site or act as carriers for siRNA and antibodies, effectively counteracting exosome-mediated immunosuppression and enhancing T-cell-mediated anti-tumor effects [84,85]. Furthermore, DNA nanostructures, acting as highly precise drug carriers, can specifically deliver thrombin to tumor-associated blood vessels, inducing intravascular thrombosis and subsequent tumor necrosis [86]. CpG-modified DNA nanostructures have also demonstrated the ability to directly activate immune cells (such as macrophages) by non-invasively entering cells and triggering endosomal immune responses, offering a novel approach to overcome tumor-associated immunosuppression and modulate pathological responses [87,88]. Researchers have developed enzyme-conjugated magnetic nanorobots that mimic the cytolytic mechanisms of human immune cells. These can be precisely guided by external magnetic fields to selectively target and eliminate breast cancer cells [89].

**Figure 9:**
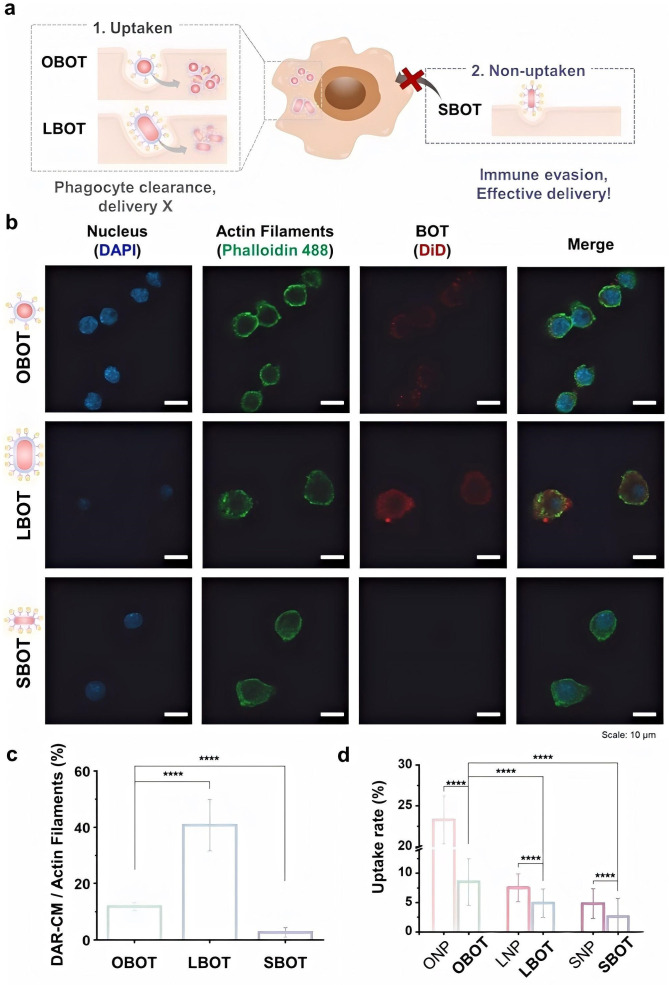
Immune clearance effect analysis results of nano-BOTs. (**a**) Schematic illustration of nano-BOT phagocyte clearance. (**b**) Cellular uptake of NPs and nano-BOTs determined by ICP-MS (mean ± standard deviation (SD), *n* = 3). (**c**) Cellular uptake observed by confocal imaging. (**d**) Mean fluorescence intensity in a single cell (BOT/actin filaments) for nano-BOTs. Data were analyzed by one-way ANOVA with Tukey’s multiple comparison test. The graphs show the mean ± SEM. Data are representative at least two independent experiments (*n* = 3/group). **** *p* < 0.0001. [95], Copyright 2025, Wiley-VCH.

Another critical role of MNRs in cancer immunotherapy is to effectively modulate the TEM by physical stimuli and structural/functional modifications [90]. MNRs can intervene in the TEM through various physical, chemical, and biological means, activating immune cells and fostering robust anti-tumor responses, ensuring that the success of immunotherapy extends beyond direct drug action to leverage a remodeled immune landscape for enhanced cancer cell recognition and elimination. MNRs impact the TEM by inducing localized heating (e.g., photothermal/magnetothermal effects), activating ultrasound, or triggering specific chemical stimuli. As shown in Figure 9, these localized interventions can effectively stimulate infiltrating immune cells (such as dendritic cells), promoting their maturation and enhanced function, which in turn induces a stronger anti-tumor immune response [90]. This approach aims to convert immunosuppressive “cold” tumors into immune-activated “hot” tumors, creating a favorable environment for immunotherapy [91,92,93,94].

### 3.6. MNRs in Combination with Emerging Treatment Strategies

**Figure 10:**
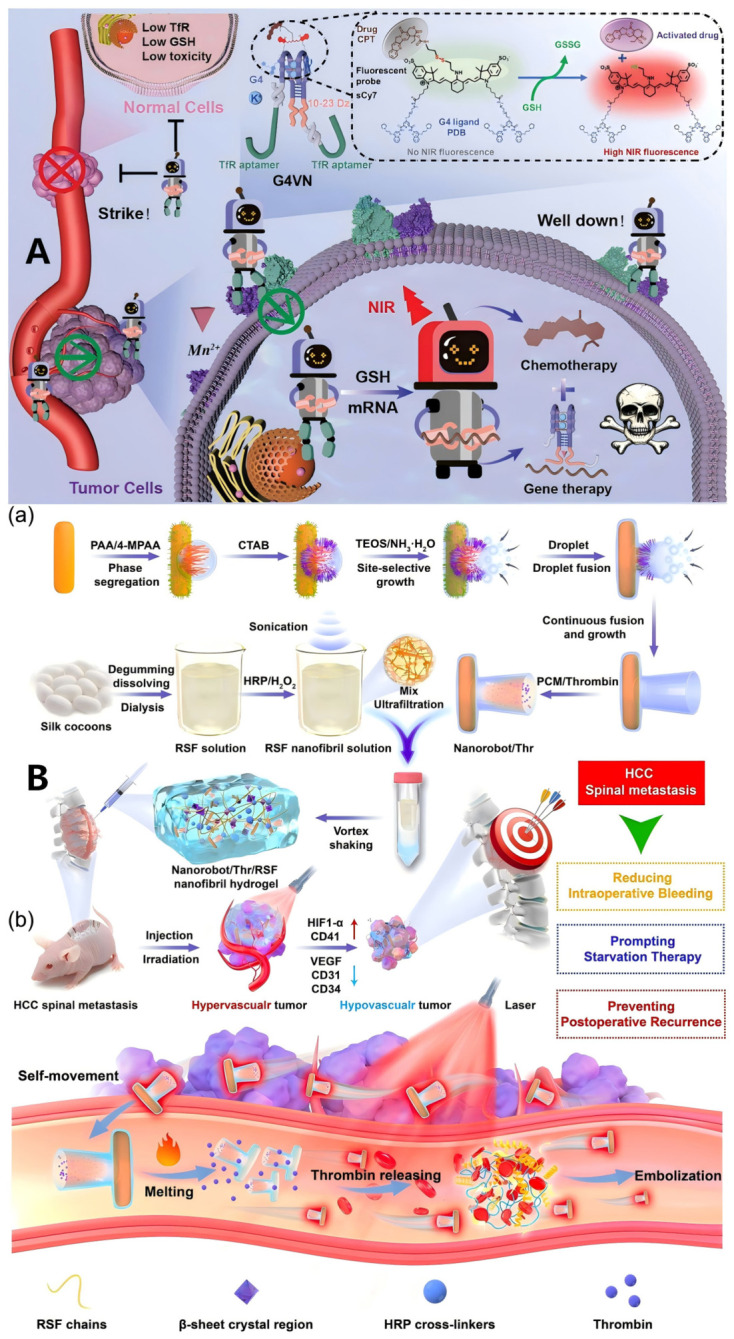
Examples of MNRs in Combination with Emerging Treatment Strategies. (**A**) Schematic for engineering the G-programmed versatile nanorobot (G4VN) for synergistic targeted therapeutics [96], Copyright 2024, Wiley-VCH; (**B**) Mechanism of action of Nanorobot/Thr/RSF nanofibril hydrogels in the treatment of HCC spinal metastasis. (**a**) Super-assembly of nanorobots, the preparation of RSF nanofibril solutions, and Nanorobots/Thr/RSF nanofibril hydrogels. (**b**) The Nanorobot/Thr/RSF nanofibril hydrogels for reducing intraoperative bleeding in HCC spinal metastasis, providing starvation embolization therapy, and preventing postoperative recurrence of HCC spinal metastasis [98], Copyright 2024, Springer Nature.

Advancements in cancer treatment research have seen the emergence of new strategies like gene editing, nanoparticle therapy, and laser therapy, which offer significant potential. Yet, these methods confront challenges such as inconsistent efficacy and poor targeting. Consequently, integrating micro/nanobots with these emerging treatment approaches is an active research frontier. As shown in Figure 10, a G-quadruplex-programmed versatile nanorobot, comprising DNAzyme and ligand–drug conjugate subunits, was proposed [96] for precise tumor targeting and concurrent gene silencing and chemotherapy. Another notable development is Zhou et al.’s [97] intelligent bimetallic MOF nanosystem, engineered for efficient gene delivery and expression. This system dually regulates intracellular acid metabolism and significantly amplifies nanocatalytic tumor therapy by enhancing apoptosis and ferroptosis. This is achieved as endogenous RNA interference and exogenous acidic substances synergistically elevate intracellular acidity, augmenting nanocatalytic ferroptosis. Moreover, the subsequent intracellular pH reduction induces calcium influx, causing mitochondrial calcium overload and thereby sensitizing cancer cells to nanocatalyst-triggered oxidative stress, apoptotic damage, and ultimately synergistic tumor suppression. In another application, nanorobot/thrombin/regenerated silk fibroin nanofibril hydrogels were utilized to mitigate intraoperative bleeding and effect starvation embolization in hepatocellular carcinoma spinal metastasis. This approach simultaneously inhibited blood supply to residual tumors and reduced neovascularization, thus preventing postsurgical recurrence [98]. Gao et al. provided a profound comprehensive summary of injectable nanorobots designed for precision cancer therapy, which focused on their dynamic performance across six critical stages: circulation, targeting, penetration, internalization, release, and treatment (CTPIRT process) [99]. Also, combination strategy involves the use of nanoparticles to simultaneously deliver chemotherapeutic agents and immunomodulatory compounds to glioma cells was used in some studies [100].

## 4. Perspectives of MNRs: Key Challenges and Pathways from Lab to Clinic

Despite the revolutionary potential of MNRs in cancer treatment, their successful translation from laboratory research to clinical application faces multiple formidable challenges. These challenges encompass not only technical aspects such as precise in vivo control and biocompatibility but also broader issues related to regulatory frameworks and clinical translation feasibility. An in-depth analysis of these bottlenecks and the exploration of potential solutions are crucial for accelerating the clinical progression of MNRs.

### 4.1. In Vivo Controllability of MNRs

Achieving precise and reliable control of MNRs within the complex and dynamic biological environment remains a primary technical hurdle for their clinical application. Current propulsion mechanisms, whether relying on external fields (e.g., magnetic fields, ultrasound, light) or internal chemical reactions, possess inherent limitations. External field-based systems, such as magnetic field-driven nanorobots, while capable of deep tissue penetration, often struggle with spatial resolution in complex tissues and require sophisticated external setups. Crucially, the strong fields necessary for effective propulsion could potentially harm surrounding healthy tissues by inducing eddy currents or local heating [101]. This necessitates that propulsion strategies must find a delicate balance between driving force, penetration depth, spatial resolution, and biological safety. Chemical propulsion systems, though generally biocompatible and self-sufficient, are notoriously difficult to precisely modulate in terms of speed and direction due to varying physiological conditions and the fluctuating availability of fuel (e.g., endogenous H_2_O_2_ and glucose). To address these challenges, future breakthroughs will depend on the development of advanced feedback control systems. Such systems must integrate real-time, high-resolution imaging technologies (e.g., ultrasound, optical imaging, and MRI) with precise actuation mechanisms, enabling MNRs to navigate through complex biological fluid flows, overcome physiological barriers like blood vessels and tissue interstices, and reach specific target sites with high accuracy [17]. Furthermore, exploring the integration of artificial intelligence and machine learning algorithms to enable autonomous path planning and adaptive control for MNRs will be vital to cope with the unpredictability of the in vivo environment.

### 4.2. Long-Term Biocompatibility of MNRs

The biocompatibility of nanomaterials, particularly their potential for cytotoxicity, immunogenicity, and the safety of long-term degradation products, presents a significant challenge in microrobotics. Traditional inorganic nanomaterials (e.g., iron oxide and gold nanoparticles), favored for their structural integrity, controllable synthesis, and diverse physicochemical properties, have been widely used in MNR construction. However, their limited biodegradability and slow clearance kinetics in vivo raise substantial toxicological concerns. Despite offering advantages in enhancing drug delivery (e.g., improved enhanced permeability and retention (EPR) effect), these inorganic components can induce inflammatory responses, fibrosis, or even long-term accumulation, leading to irreversible tissue damage [54]. To effectively mitigate these issues and enhance the functionality of nanorobots in complex biological environments, the integration of bio-inspired components has emerged as a key trend. Incorporating natural biomaterials such as cells, organelles, proteins, or enzymes into MNR designs not only significantly boosts biocompatibility and reduces immunogenicity but also allows for propulsion strategies using biocompatible chemical fuels or external fields, thereby minimizing off-target effects and systemic toxicity. A pivotal advancement in this regard is cell membrane biomimetic technology, wherein synthetic nanorobots are coated with biological membranes to achieve sophisticated biological camouflage. This strategy confers several advantages: first, immune evasion through surface signals like CD47, enabling prolonged systemic circulation and enhanced tumor penetration [102]; second, homologous targeting facilitated by parent cell-specific antigens on cancer cell membranes; and finally, the integration of biological functions, such as targeting inflammation and thrombosis via platelet membranes [103,104]. These cytomembrane-camouflaged or immunocyte-integrated MNRs, typically prepared by direct membrane coating or phagocytosis [105], not only effectively evade phagocytosis but also gain enhanced propulsion in biofluids, targeted delivery to diseased areas, and the neutralization of bacterial toxins. However, a comprehensive evaluation of the long-term in vivo degradation pathways, metabolic products, and potential chronic immune reactions (e.g., cytokine storms or autoimmune responses) induced by these biomimetically protected MNRs remains largely unexplored. This necessitates more rigorous and long-term toxicological and pharmacokinetic studies.

### 4.3. Regulatory Barriers of MNRs

Integrating micro/nanorobots into clinical practice faces considerable regulatory hurdles, primarily stemming from their inherent novelty and complexity. Unlike conventional drugs or medical devices, these autonomous or semi-autonomous biological–technological hybrids do not fit neatly into existing regulatory frameworks. MNRs may possess attributes of drugs (e.g., carrying active payloads), devices (e.g., propulsion and sensing capabilities), and biologics (e.g., incorporating living cells or biomembranes), thus necessitating an urgent establishment of clear classification criteria. The current absence of definitive guidance for evaluating the safety and efficacy of innovative medical devices, especially those featuring active biological components or self-propelling capabilities, poses a significant obstacle for developers. Therefore, the international harmonization of regulatory standards is imperative, as it will not only streamline the approval process but also facilitate the global adoption and widespread therapeutic application of MNRs. There is an urgent need to establish specific testing methodologies, manufacturing standards, and robust post-market surveillance protocols tailored for MNRs to ensure their safety and efficacy throughout their lifecycle.

### 4.4. Clinical Translation Feasibility of MNRs

Despite encouraging preclinical outcomes, the clinical translation of micro/nanorobots is impeded by several practical challenges. A primary concern is manufacturing scalability. Current MNR fabrication techniques are often labor-intensive and produce limited quantities, which significantly complicates large-scale production for clinical trials and commercialization. Achieving the leap from laboratory-scale batch preparation to industrial-scale production requires the development of more efficient, automated, and cost-effective manufacturing processes. Secondly, the cost-effectiveness of these advanced technologies must be rigorously demonstrated, particularly when benchmarked against established therapeutic alternatives. High research, development, and production costs could be a major barrier to their widespread adoption; therefore, strategies to reduce costs, such as modular design, standardized production processes, and the utilization of novel materials and manufacturing techniques, need to be explored. Finally, physician and patient acceptance will hinge on multiple factors, including the ease of use of MNRs, their evident clinical benefits, and the ethical implications associated with deploying autonomous agents within the human body. Successful clinical adoption will necessitate seamlessly integrating MNRs into existing clinical workflows, developing intuitive user interfaces for physician control, and proactively addressing intellectual property issues. Therefore, future development not only requires technological breakthroughs but also demands attention to their social, economic, and ethical sustainability to accelerate the benefits of MNRs to a wider patient population.

## 5. Conclusions

Over the past decade, micro/nanorobots (MNRs) have catalyzed significant breakthroughs across various domains of precision medicine. This rapid progress is largely attributable to their ultraminiature dimensions and untethered nature—traits that grant them unprecedented maneuverability and functional versatility within confined biological microenvironments. Functionally, these platforms span a broad spectrum of biomedical applications. They operate as sophisticated diagnostic biosensors capable of mapping in vivo ion concentrations; serve as dynamic agents for advanced optical, acoustic, and magnetic imaging methodologies; and function as highly specific delivery vehicles engineered to transport pharmacological payloads, biological macromolecules, or even living cells. Moreover, in microsurgical contexts, MNRs exhibit remarkable capability in probing delicate tissues and selectively eradicating malignant cells or bacterial pathogens.

Despite extensive validation of these targeted interventions in diverse animal models, the clinical translation of MNRs remains obstructed by a complex nexus of safety, technical, regulatory, and commercial barriers. Most notably, direct application in human subjects has yet to be initiated, meaning the long-term systemic toxicity and overall biological impact of these nanostructures are still largely uncharacterized. Parallel to these safety concerns, the physical actuation and propulsion mechanisms governing MNR navigation demand substantial optimization to guarantee reliable, autonomous control deep within the human body.

Nevertheless, while the trajectory toward large-scale clinical integration is fraught with challenges, the transformative capacity of these microrobotic systems in disease diagnosis and therapy is undeniable. Should preliminary human trials successfully demonstrate their clinical viability, particularly their ability to enable precision interventions, significantly alleviate surgical trauma, and curtail healthcare costs. MNRs will invariably accelerate the evolution of modern biomedicine and fundamentally reshape human healthcare paradigms.

## Figures and Tables

**Table 1:** MNRs in cancer therapy based on inorganic materials.

Material Type	Specific Material Examples	Propulsion Mode	Therapeutic Functions
Inorganic Materials	Pt; MnO_2_; ZnO; Gold Nanorods; Fe; Fe_3_O_4_; Ga; ZnS; TiO_2_; Copper Sulfide; Graphene; Polyaniline.	Chemical Propulsion	Targeted drug delivery; Localized tumor destruction; Real-time monitoring.
		Magnetic Propulsion	Precise remote navigation; Blood–brain barrier penetration; MRI contrast enhancement; Theranostic integration; Drug delivery; Tumor growth inhibition.
		Light Propulsion	Cell-level manipulation; Photothermal Therapy (PTT); Photodynamic Therapy (PDT); Targeted drug delivery; Tumor growth inhibition; Immune modulation.
		Ultrasound Propulsion	Targeted drug delivery; Enhanced cell membrane permeability; Acoustic imaging; Sonopermeation; Antibacterial activity; Promotion of cancer cell endocytosis.

**Table 2:** MNRs in cancer therapy based on organic/polymeric materials.

Material Type	Specific Material Examples	Propulsion Mode	Therapeutic Functions
Organic/ Polymeric Materials	Heparin/Folic Acid/L-Arginine (HFLA); Poly(N-isopropylacrylamide) (PNIPAM); Polymer Matrices (Ecoflex, Polydimethylsiloxane, Hydrogel); Poly(butyl cyanoacrylate) (PBCA); Poly(methyl methacrylate) (PMMA); Polyvinyl Alcohol (PVA); Polyvinylpyrrolidone (PVP); Poly(lactic-co-glycolic acid) (PLGA); Poly(vinyl acetate) (PVAX).	Chemical Propulsion	Targeted drug delivery; Reversal of multidrug resistance; Localized tumor destruction.
		Ultrasound Propulsion	Drug delivery; Therapeutic gas release; Blood-brain barrier penetration; Sonopermeation; Antibacterial activity; Enhanced ultrasound imaging.

**Table 3:** MNRs in cancer therapy based on biological/hybrid materials.

Material Type	Specific Material Examples	Propulsion Mode	Therapeutic Functions
Biological/Hybrid Materials	Magnetotactic Bacteria; Salmonella; Sperm; Neutrophils; Red Blood Cells; Virus-Like Particles (VLPs); DNA Origami Structures; Urease; Acetylcholinesterase; Glucose oxidase; Aldolase.	Internal Field (Bio-Hybrid Driving)	Targeted drug delivery; Localized tumor cell destruction; Tumor tissue sampling; Real-time monitoring; Reversal of multidrug resistance; Immune modulation; Inhibition of tumor growth and recurrence; Alleviation of surgical trauma.
		External Field (External-Field Hybrid Driving)	Cell-level manipulation; Photothermal Therapy (PTT); Photodynamic Therapy (PDT); Targeted drug delivery; Immune modulation.

## Data Availability

No new data were created or analyzed in this study.
